# Editorial: Risks and Benefits of Adjuvants to Cancer Therapies

**DOI:** 10.3389/fonc.2022.913626

**Published:** 2022-05-16

**Authors:** Peixin Dong, David A. Gewirtz

**Affiliations:** ^1^ Department of Obstetrics and Gynecology, Hokkaido University School of Medicine, Hokkaido University, Sapporo, Japan; ^2^ Department of Pharmacology & Toxicology, Massey Cancer Center, Virginia Commonwealth University, Richmond, VA, United States; ^3^ Department of Medicine, Massey Cancer Center, Virginia Commonwealth University, Richmond, VA, United States

**Keywords:** Cancer therapy, adjuvant therapy, vitamin E, herbal, probiotics, curcumin, alantolactone, thalidomide

Cancer patients may have minimal residual disease after completing primary treatment, which could be a source of subsequent early recurrence and metastasis. Adjuvant therapy (including chemotherapy, radiation therapy, hormone therapy, targeted therapy, and immunotherapy) has the potential to remove minimal residual disease and increase patient survival. In contrast, neoadjuvant therapy is utilized to shrink tumors prior to the primary treatment. Ongoing research in the fields of adjuvant and neoadjuvant cancer therapy is expected to yield new drugs and innovative approaches that can be combined with existing therapies to improve patient outcomes and prevent cancer recurrence.

The current Research Topic, titled “Risks and Benefits of Adjuvants to Cancer Therapies” includes 12 scientific studies (original research articles, reviews, and case reports).

Khallouki et al. established that vitamin E compounds, known as tocols (including tocopherols and tocotrienols), directly interact with estrogen receptors (ERs), thereby activating the transcription of an estrogen-responsive reporter gene in breast cancer cells. Tocopherols induce the proliferation of ER-positive breast cancer cells but not ER-negative breast cancer cells, while tocotrienols inhibit the proliferation of both ER-positive and ER-negative breast cancer cells. These studies indicate that tocopherols and tocotrienols have different roles in regulating cancer cell proliferation.

Zha et al. assessed the benefits and hazards of postoperative adjuvant chemotherapy versus surgery alone in patients with colorectal cancer. They found that patients with stage II/III colorectal cancer can benefit greatly from postoperative adjuvant chemotherapy, providing useful information for making decisions about the advantages and hazards of adjuvant chemotherapy in patients with colorectal cancer following resection.

Mei et al. investigated whether adjuvant treatment would benefit patients with pT2N0M0 gastric cancer, which is defined as tumors infiltrating the muscularis propria [T2], no regional lymph node metastases [N0], and no distant metastasis [M0]. Patients with pT2N0M0 gastric cancer who received adjuvant chemotherapy had higher 5-year overall survival and disease-specific survival rates. Thus, adjuvant chemotherapy may be considered for patients with pT2N0M0 gastric cancer.

Fasinu and Rapp reviewed the interaction between herbal and chemotherapeutic drugs. According to recent patient data, some of these supplements may interact with chemotherapy drugs. As a result, it would be prudent to avoid taking anti-cancer medications and natural products at the same time.

Lu et al. reviewed the effects of probiotics in preventing and treating cancer. As gastrointestinal discomfort is a common side effect of anti-tumor therapy, probiotics can help to improve the intestinal environment, increase the functionality of the intestinal mucosal barrier, and minimize the occurrence of diarrhea. The ability of probiotics to improve anti-cancer side effects has been also linked to innate immunity.

Xu et al. summarized the potential clinical applications of curcumin as an adjuvant to osteosarcoma treatment. Even though curcumin appears to have a high synergistic effect in other therapies (chemotherapy, immunotherapy, bone tissue engineering, and biomaterials), curcumin’s properties such as hydrophobicity and low absorption, hinder its anticancer impact. Clearly, more research will be required to resolve these challenges.

Tian et al. discussed the advantages of carboplatin- and paclitaxel-based adjuvant and neoadjuvant chemotherapies in early triple-negative breast cancer. Their review shows that in both neoadjuvant and adjuvant contexts, the combination of carboplatin and paclitaxel resulted in a greater histological complete response in patients with early triple-negative breast cancer.

Cai et al. reviewed the molecular processes by which Alantolactone, a natural chemical isolated from the Chinese traditional medicine Inula helenium L, exerts anti-cancer effects and the potential of alantolactoneas as cancer therapeutic agents.

Xie et al. conducted a comprehensive assessment of the efficacy and safety of thalidomide in the treatment of chemotherapy-induced nausea and vomiting (CINV) in patients who had received highly emetogenic chemotherapy (HEC). The authors pointed out that thalidomide is effective and safe for preventing CINV in HEC patients, and that it has a considerable propensity to improve patients’ quality of life.

Jiang et al. explored the connection between antibiotic use and the survival of cancer patients receiving immune checkpoint inhibitors. The authors proposed that antibiotic administration was significantly associated with worse progression-free survival and overall survival in these patients. Consequently, antibiotics should likely be used with caution in cancer patients who are being treated with immune checkpoint inhibitors.

In a case report by Money et al., the use of intravenous administration of magnesium before cisplatin was found to be the best practice to prevent cisplatin-induced acute kidney injury and hypomagnesemia. This is an intriguing observation that is likely deserving follow-up in the clinic.

Takayama et al. reported on a case of advanced malignant melanoma that developed prolonged anorexia and nausea after receiving nivolumab and was successfully treated with Kampo medications.

Overall, these papers in this Frontiers Research Topic touch on recent efforts to uncover novel adjuvant cancer medications, as well as new combinations of adjuvant therapies with existing treatments. For many malignancies, recommending adjuvant therapy and selecting the best medicines remains a challenge. Because human malignancies have such a wide range of remarkable biological diversity that affects treatment efficacy, optimal management or precision adjuvants will be required to generate effective therapeutic strategies where the anticipated benefits will have to be balanced with issues of tolerability. Promising prognostic and predictive biomarkers may help guide adjuvant therapy use (**Figure 1**).

**Figure 1:**
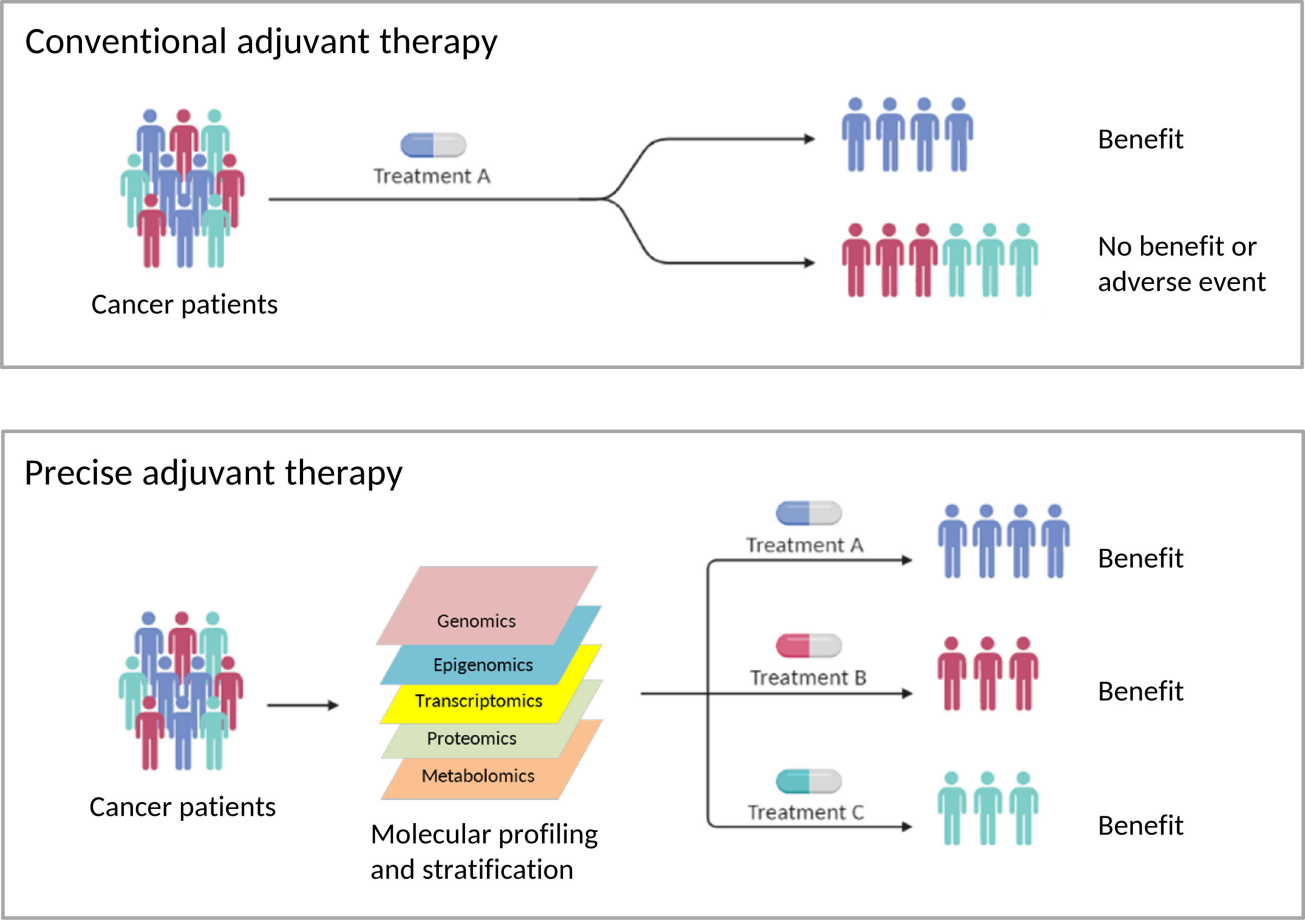
The Risks and Benefits of Adjuvants to Cancer Therapies (created with BioRender.com). Conventional adjuvant therapies aim to provide treatments for the average cancer patient. As a result, this strategy is less effective in treating individual patients, and it is difficult to avoid side effects. To develop successful adjuvant therapies, optimal management or precise adjuvants will be necessary, where the predicted advantages must be balanced with concerns about tolerance. Promising prognostic and predictive biomarkers might help modify existing adjuvant cancer therapies or lead to the discovery of new adjuvant cancer therapies or adjuvant therapy combinations.

## Author Contributions

All authors listed have made a substantial, direct, and intellectual contribution to the work and approved it for publication.

## Funding

PD was supported by a grant from JSPS Grant-in-Aid for Scientific Research (C) (19K09769; 22K09541 and 22K09634). Work in the Gewirtz laboratory is supported by NIH/NCI grants CA260819 and CA239706 and by Grant #W81XWH19-1-0490 from the Department of Defense Breast Cancer Research Program.

## Conflict of Interest

The authors declare that the research was conducted in the absence of any commercial or financial relationships that could be construed as a potential conflict of interest.

## Publisher’s Note

All claims expressed in this article are solely those of the authors and do not necessarily represent those of their affiliated organizations, or those of the publisher, the editors and the reviewers. Any product that may be evaluated in this article, or claim that may be made by its manufacturer, is not guaranteed or endorsed by the publisher.

